# Short-acting insulin analogues versus regular human insulin on postprandial glucose and hypoglycemia in type 1 diabetes mellitus: a systematic review and meta-analysis

**DOI:** 10.1186/s13098-018-0397-3

**Published:** 2019-01-03

**Authors:** Karla F. S. Melo, Luciana R. Bahia, Bruna Pasinato, Gustavo J. M. Porfirio, Ana Luiza Martimbianco, Rachel Riera, Luis E. P. Calliari, Walter J. Minicucci, Luiz A. A. Turatti, Hermelinda C. Pedrosa, Beatriz D. Schaan

**Affiliations:** 1 Diabetes Division, Hospital de Clínicas, School of Medicine, Universidade de São Paulo, São Paulo, Brazil; 2 Sociedade Brasileira de Diabetes, Rua Afonso Brás, Rua Afonso Brás, 579, cjs 72/74, Vila Nova Conceição, 04511-011 São Paulo, SP Brazil; 3 Quasar Telemedicina Ltda, São Paulo, Brazil; 4 Universidade Estadual do Rio de Janeiro, Rio de Janeiro, RJ Brazil; 5 Department of Internal Medicine, School of Medicine, Universidade Federal do Rio Grande do Sul, Porto Alegre, Brazil; 6 Cochrane Brazil, São Paulo, Brazil; 7 School of Medicine, Universidade Federal de São Paulo, São Paulo, Brazil; 8 Pediatric Endocrine Unit, Pediatric Department, Santa Casa School of Medical Sciences, São Paulo, Brazil; 9 Endocrine Division, Hospital de Clínicas de Porto Alegre, Porto Alegre, Brazil

**Keywords:** Diabetes mellitus, Type 1 diabetes, Insulin, Aspart insulin, Glulisine insulin, Lispro insulin, Systematic review, Meta-analysis

## Abstract

**Introduction:**

Strict glucose control using multiple doses of insulin is the standard treatment for type 1 diabetes mellitus (T1DM), but increased risk of hypoglycemia is a frequent drawback. Regular insulin in multiple doses is important for achieving strict glycemic control for T1DM, but short-acting insulin analogues may be better in reducing hypoglycemia and postprandial glucose levels.

**Objective:**

We conducted a systematic review and meta-analysis of randomized controlled trials (RCTs) to assess the effects of short-acting insulin analogues *vs* regular human insulin on hypoglycemia and postprandial glucose in patients with T1DM.

**Methods:**

Searches were run on the electronic databases MEDLINE, Cochrane-CENTRAL, EMBASE, ClinicalTrials.gov, LILACS, and DARE for RCTs published until August 2017. To be included in the study, the RCTs had to cover a minimum period of 4 weeks and had to assess the effects of short-acting insulin analogues *vs* regular human insulin on hypoglycemia and postprandial glucose levels in patients with T1DM. Two independent reviewers extracted the data and assessed the quality of the selected studies. The primary outcomes analyzed were hypoglycemia (total episodes, nocturnal hypoglycemia, and severe hypoglycemia) and postprandial glucose (at all times, after breakfast, after lunch, and after dinner). Glycated hemoglobin (HbA1c) levels and quality of life were considered secondary outcomes. The risk of bias of each RCT was assessed using the Cochrane Collaboration Risk of Bias table, while the quality of evidence for each outcome was assessed using the GRADEpro software. The pooled mean difference in the number of hypoglycemic episodes and postprandial glucose between short-acting insulin analogues vs. regular human insulin was calculated using the random-effects model.

**Results:**

Of the 2897 articles retrieved, 22 (6235 patients) were included. Short-acting insulin analogues were associated with a decrease in total hypoglycemic episodes (risk rate 0.93, 95% CI 0.87–0.99; 6235 patients; I^2^ = 81%), nocturnal hypoglycemia (risk rate 0.55, 95% CI 0.40–0.76, 1995 patients, I^2^ = 84%), and severe hypoglycemia (risk rate 0.68, 95% CI 0.60–0.77; 5945 patients, I^2^ = 0%); and with lower postprandial glucose levels (mean difference/MD − 19.44 mg/dL; 95% CI − 21.49 to − 17.39; 5031 patients, I^2^ = 69%) and lower HbA1c (MD − 0,13%; IC 95% − 0.16 to − 0.10; 5204 patients; I^2^ = 73%) levels.

**Conclusions:**

Short-acting insulin analogues are superior to regular human insulin in T1DM patients for the following outcomes: total hypoglycemic episodes, nocturnal hypoglycemia, severe hypoglycemia, postprandial glucose, and HbA1c.

**Electronic supplementary material:**

The online version of this article (10.1186/s13098-018-0397-3) contains supplementary material, which is available to authorized users.

## Introduction

Hyperglycemia caused by diabetes mellitus is associated with long-term diabetes-related complications, resulting in reduced life expectancy when compared to the general population without diabetes [1]. In type 1 diabetes mellitus (T1DM), increased mortality is explained by diabetic ketoacidosis and hypoglycemia early in life and cardiovascular diseases later in life. Strict glucose control is associated with a lower risk of diabetes-related complications and cardiovascular mortality [2, 3]. Besides sustained chronic hyperglycemia, another particular atherogenic action of postprandial glucose has emerged as another target to be pursued in the clinical practice aimed at reducing mean blood glucose and glycated hemoglobin (HbA1c) [4]. Lower postprandial glucose levels may be associated with a lower risk of cardiovascular outcomes in diabetes [5].

However, strict glucose control is associated with weight gain and a higher incidence of hypoglycemia [2]. Hypoglycemia can lead to seizures, cognitive impairment, decreased quality of life, loss of work productivity, impaired functioning on the following day, and non-adherence to treatment [6–10]. Hypoglycemia may also cause cardiac ischemia or arrhythmia mediated by the catecholamine secretion [11], eventually leading to a higher risk of death [12].

The development of insulin analogues through molecular structure modifications of human insulin is based on pharmacokinetic profiles that try to mimic the physiological secretion of insulin [13]. Short-acting insulin analogues (aspart, glulisine, and lispro) are thought to be better than regular human insulin due to faster absorption and faster onset of action, mimicking better the physiological prandial insulin peak of people without diabetes [14, 15] and leading to lower postprandial glucose levels [16]. Potentially, this allows for better glucose control, reduces the number of hypoglycemic episodes, and helps improve the patient’s quality of life by allowing for less restrictive mealtimes. However, a number of meta-analyses on short-acting insulin analogues have found only modest benefits on glucose control and the frequency of hypoglycemic episodes compared to therapy with regular human insulin [17–19]. Analyzing the expected benefits and higher costs of insulin analogues against the cost-effectiveness of human insulin is particularly important in low- to middle-income countries, where the lack of affordable insulin medication is still a major cause of death [20].

It remains unclear whether short-acting insulin analogues are indeed superior to regular human insulin in reducing hypoglycemia and lowering postprandial glycemia. The aim of the present systematic review and meta-analysis was to evaluate the outcomes (all hypoglycemic episodes, nocturnal and severe hypoglycemia, and postprandial glycemia) associated with the use of short-acting insulin analogues in T1DM as compared to regular human insulin.

## Methods

This systematic review was carried out based on the methodology described in the Cochrane Collaboration tool [21].

### Eligibility criteria

Studies were eligible for inclusion if they were randomized controlled trials (RCTs), included children and adults with a diagnosis of T1DM for at least 1 year, with or without chronic complications, and compared the use of subcutaneous short-acting insulin analogues (aspart, glulisine, and lispro) with regular human insulin for at least 4 weeks. The primary outcomes were hypoglycemia (all hypoglycemic episodes, nocturnal hypoglycemia, and severe hypoglycemia) and postprandial glucose (all meals and after breakfast, lunch, and dinner). Secondary outcomes included long-term glucose control assessed by HbA1c and changes in quality of life. Studies with pregnant women, patients with acute diabetic decompensation, or patients with type 2 diabetes, studies that used insulin pumps, experimental studies or retrospective studies, narrative reviews, letters, and congress abstracts were excluded.

### Information sources

We searched the following electronic databases for studies published until August 2017: MEDLINE (via PubMed), EMBASE (via Elsevier), CENTRAL (the Cochrane Central Register of Controlled Trials), LILACS (Literatura Latino-Americana e do Caribe em Ciências da Saúde, via BVS), and DARE (Database of Abstracts of Reviews of Effects). The references cited by all the relevant studies were hand searched. We performed an additional search for ongoing and/or unpublished studies in the US National Institute of Health Ongoing Trials Register (http://www.clinicaltrials.gov) and the International Clinical Trials Registry Platform (ICTRP - WHO). No language restrictions were applied.

### Search strategy

We searched for the terms ‘diabetes mellitus, type 1’, ‘aspart’, ‘glulisine’, ‘lispro’, and related terms to obtain as many results as possible. The complete search strategies used for each database are provided as Additional file 1: Table S1.

### Study selection

Duplicates were manually identified and excluded. The articles were then analyzed in two steps: firstly, two reviewers independently screened the titles and abstracts yielded by the search strategy against the inclusion and exclusion criteria; secondly, the same reviewers independently screened the full text reports and selected the articles that met the inclusion criteria. Disagreements were resolved by consensus. If no agreement could be reached, a third reviewer was consulted for arbitration. Agreement between reviewers was assessed using Cohen’s kappa coefficient. The Rayyan software (Rayyan Platform) was used for this selection process.

### Data extraction and quality assessment

Two reviewers independently extracted data from each study using an extract table template, which provided the following information: title of the study; demographic characteristics; study design; intervention details; and outcomes. A third reviewer further assessed all RCTs to check for completeness of data.

In case of missing data, the authors of the studies were contacted for additional information. If the missing data could not be retrieved, the study was not included. Retrieved missing data were presented in a narrative form.

To assess the internal quality of the studies, we ran each RCT through the Cochrane Collaboration tool for assessing the risk of bias [21]. The following potential issues were assessed: random sequence generation; allocation concealment; blinding of participants and outcome assessors; blinding of outcomes; incomplete outcome data; selective reporting; and other sources of bias. For each domain, the risk of bias was rated as low, high, or unclear. The quality of evidence was assessed using the GRADEpro GDT software (GRADEpro 2014). The results were presented in a “Summary of Findings” table.

### Data synthesis and analysis

Analyses were conducted using the RevMan 5.3 software. Relative risk was used as a summary measure of effect size for dichotomous outcomes, the mean difference was used for continuous outcomes, and the risk rate was used for outcomes related to the number of events. The meta-analysis was performed using a random-effects model based on the DerSimonian–Laird method, 95% confidence interval (95% CI). A *p*-value < 0.1 was considered statistically significant. Heterogeneity between the studies was assessed using I^2^ statistic, in which values above 50% were indicative of high heterogeneity [21]. Heterogeneity as determined by the Chi square test was considered non-significant for I^2^ values between 0 and 50%, moderate for values between 51 and 79%, and significant for values between 80 and 100%. Where possible, study data were pooled and summarized in meta-analysis charts (quantitative synthesis) using the RevMan 5.3 software; otherwise, the results of each study were presented individually (qualitative synthesis).

### Subgroup and sensitivity analysis

Subgroup analyses were designed based on the effects by neutral protamine Hagedorn (NPH) human insulin vs. long-acting insulin analogues. We also carried out a sensitivity analysis for the primary outcome ‘all hypoglycemic episodes’ considering the risk of bias in the studies. A second meta-analysis was then performed for this outcome excluding studies with three or more domains classified as “high risk of bias”.

## Results

### Literature search

The electronic search yielded a total of 2897 references. We found 1761 references in MEDLINE, 670 in EMBASE, 216 in CENTRAL, 110 in ClinicalTrials.gov, and none in the International Clinical Trials Registry Platform (ICTRP-WHO). We then removed duplicates (328) and selected the studies as shown in Additional file 2: Figure S1.

### Description of studies

In the initial search, 2569 potentially relevant citations were retrieved, of which 22 articles met the inclusion criteria. Eight RCTs analyzed the effects of aspart vs. regular human insulin [35–42], one analyzed the effects of glulisine vs. regular human insulin [43], and 13 analyzed the effects of lispro vs. regular human insulin [22–34].The characteristics of each study are described in Table 1.

**Table 1:** Characteristics of the included studies

Study/year	Design	N	Age (years)	Time with DM (mean, years)	Comparison	Basal insulin	Treatment time	Outcomes
Anderson et al. (1997)	Open, multicenter, crossover	336	32.1 (mean)	12	LISPRO (right before meal) × REGULAR (30–45 min before meal)	NPH and Long-acting insulin analogue (Humulin U)	12 months	Total hypoglycemia Postprandial glucose Glycated hemoglobin
Annuzzi et al. (2001)^a^	Multicenter, crossover	85	31 (mean)	12	LISPRO (right before meal) × REGULAR (30-45 min before meal)	NPH	3 months	Total hypoglycemia Severe hypoglycemia Glycated hemoglobin Quality of life
Brock Jacobsen et al. (2011)	Double blind, single center, crossover	16	From 18 to 60	1	ASPART × REGULAR (both right before meal)	NPH	2 months	Total hypoglycemia Nocturnal hypoglycemia Postprandial glucose Glycated hemoglobin Quality of life
Cherubini et al. (2006)	Open, single center, parallel	30	8.1 (mean)	5.2	ASPART (2 min before meal) × REGULAR (30 min before meal). Counting of carbohydrates	Glargine	4.5 months	Total hypoglycemia Postprandial glucose Glycated hemoglobin
Danne et al. (2007)	Open, multicenter, crossover	26	5 (mean)	1.8	ASPART (right before meal) × REGULAR (30 min before meal)	NPH	3 months	Total hypoglycemia Severe hypoglycemia Patient satisfaction
Fairchild et al. (2000)	Open, single center, crossover	35	From 5 to 10	3.1	LISPRO (right before meal) × REGULAR (30 min before meal)	NPH	3 months	Total hypoglycemia Nocturnal hypoglycemia Severe hypoglycemia Postprandial glucose Glycated hemoglobin
Ferguson et al. (2001)	Open, single center, crossover	33	46 (mean)	26	LISPRO (right before meal) × REGULAR (30 min before meal)	NPH	4.5 months	Total hypoglycemia Nocturnal hypoglycemia Severe hypoglycemia Postprandial glucose Glycated hemoglobin Quality of life
Ford-Adams et al. (2003)	Multicenter, crossover	23	From 7 to 11	Not provided	LISPRO × REGULAR	NPH	4 months	Total hypoglycemia Nocturnal hypoglycemia Glycated hemoglobin
Gale et al. (2000)	Double blind, multicenter, crossover	93	35 (median)	13 (median)	LISPRO × REGULAR (both right before meal)	NPH	3 months	Total hypoglycemia Nocturnal hypoglycemia Severe hypoglycemia Postprandial glucose Glycated hemoglobin Quality of life
Garg et al. (2005)	Open, multicenter, parallel	860	40.3 (mean)	20	GLULISINE (0-15 min before meal) × REGULAR (30–45 min before meal)	Glargine	3 months	Total hypoglycemia Nocturnal hypoglycemia Severe hypoglycemia Postprandial glucose Glycated hemoglobin
Heller et al. (1999)	Open, multicenter, crossover	165	38 (mean)	16	LISPRO (right before meal) × REGULAR (30 min before meal)	NPH	4 months	Total hypoglycemia Nocturnal hypoglycemia Severe hypoglycemia Postprandial glucose Glycated hemoglobin
Heller et al. (2004)	Double blind, multicenter, crossover	155	35.7 (mean)	2	ASPART × REGULAR (both right before meal)	NPH	4 months	Total hypoglycemia Severe hypoglycemia Nocturnal hypoglycemia Glycated hemoglobin
Holcombe et al. (2002)	Open, multicenter, crossover	463	14.9 (mean)	6	LISPRO (right before meal) × REGULAR (30–45 min before meal)	NPH	4 months	Total hypoglycemia Nocturnal hypoglycemia Severe hypoglycemia Postprandial glucose Glycated hemoglobin Adverse event
Holleman et al. (1997)	Open, multicenter, crossover	199	35.4 (mean)	13	LISPRO (right before meal) × REGULAR (30 min before meal)	NPH	3 months	Total hypoglycemia Nocturnal hypoglycemia Severe hypoglycemia Postprandial glucose Glycated hemoglobin Life quality
Home et al. (1998)	Multicenter, crossover	104	34 (mean)	15	ASPART X REGULAR (both right before meal)	NPH	1 month	Total hypoglycemia Severe hypoglycemia Postprandial glycemia Glycated hemoglobin
Home et al. (2000), Bott et al. (2003), Home et al. (2006)	Open, multicenter, parallel	1070	38 (mean)	15	ASPART (right before meal) × REGULAR (30 min before meal)	NPH	6 months	Total hypoglycemia Severe hypoglycemia Postprandial glucose Glycated hemoglobin Life quality
Jacobs et al. (1997)	Open, multicenter, crossover	12	18 (mean)	Not provided	LISPRO (right before meal) × REGULAR (30 min before meal)	NPH	1 month	Total hypoglycemia Nocturnal hypoglycemia Postprandial glucose Glycated hemoglobin
Provenzano et al. (2001)	Open, multicenter, crossover	12	28 (mean)	12	LISPRO × REGULAR (both right before meal)	Long-acting insulin	168 days	Total hypoglycemia Nocturnal hypoglycemia Postprandial glucose Glycated hemoglobin
Raskin et al. (2000)	Open, multicenter, parallel	882	39.2 (mean)	1.5	ASPART (right before meal) × REGULAR (30 min before meal)	NPH	6 months	Total hypoglycemia Severe hypoglycemia Glycated hemoglobin
Tamas et al. (2001)	Open, multicenter, parallel	423	From 18 to 70	14	ASPART (0 to 5 min before meal) × REGULAR (30 min before meal)	NPH	16 months	Total hypoglycemia Severe hypoglycemia Postprandial glucose Glycated hemoglobin Life quality
Tupola et al. (2001)	Open, multicenter, crossover	29	6 (mean)	3	LISPRO (30 min after the patient started eating) × REGULAR (20 to 39 min before meal)	NPH	3 months	Total hypoglycemia Glycated hemoglobin
Vale et al. (2001)	Open, multicenter, parallel	1.184	38.7 (mean)	19.4	LISPRO (right before meal) × REGULAR (30 min before meal)	NPH	3 months	Total hypoglycemia Severe hypoglycemia Postprandial glucose Glycated hemoglobin

^a^Data from this study were taken from Siebenhofer et al. [51]

Most studies (77.2%) were multicenter trials, and the countries with the largest participation were the United States, United Kingdom, Australia, Italy, Germany, Canada, Denmark, and Finland. All studies were published between 1996 and 2011.

The selected studies contributed to a combined sample of 6235 patients for the meta-analysis. Sample sizes varied between the studies, with a minimum of 12 [23, 30] and a maximum of 1184 patients [32]. The mean age of the participants ranged from 5 to 60 years, with five studies including only children and adolescents [26, 31, 33, 34, 39]. The time since the diagnosis of T1DM ranged from 1 to 20 years.

NPH insulin was the most widely used type of basal insulin. Duration of treatment ranged between 1 and 16 months. RCTs aimed at assessing metabolic stabilization and drug adaptation carried out a run-in period that lasted up to 2 months. In most articles, short-acting insulin analogues were administered immediately before meals, and regular human insulin was administered 30–45 min before meals.

### Assessment of the risk of bias

The risk of bias was assessed using the Cochrane Collaboration tool [21]. The domains with the highest risk of bias were the lack of patient and research team blinding for the treatments and the assessment of subjective outcomes. Results of the assessment and the percentage distribution of the risk of bias by domain are shown in Additional file 3: Figure S2, Additional file 4: Table S2 and Additional file 6: Table S3.

### Hypoglycemia

#### All hypoglycemic episodes

All 22 studies included in this meta-analysis had information on the number of all hypoglycemic episodes per month and were thus included in our count [22–43]. Short-acting insulin analogues (aspart, glulisine, and lispro) were not associated with a lower number of all hypoglycemic episodes per month when compared to regular human insulin (risk rate 0.94, 95% CI 0.89–1.00; 6235 patients; I^2^ = 80%).

**Fig. 1:**
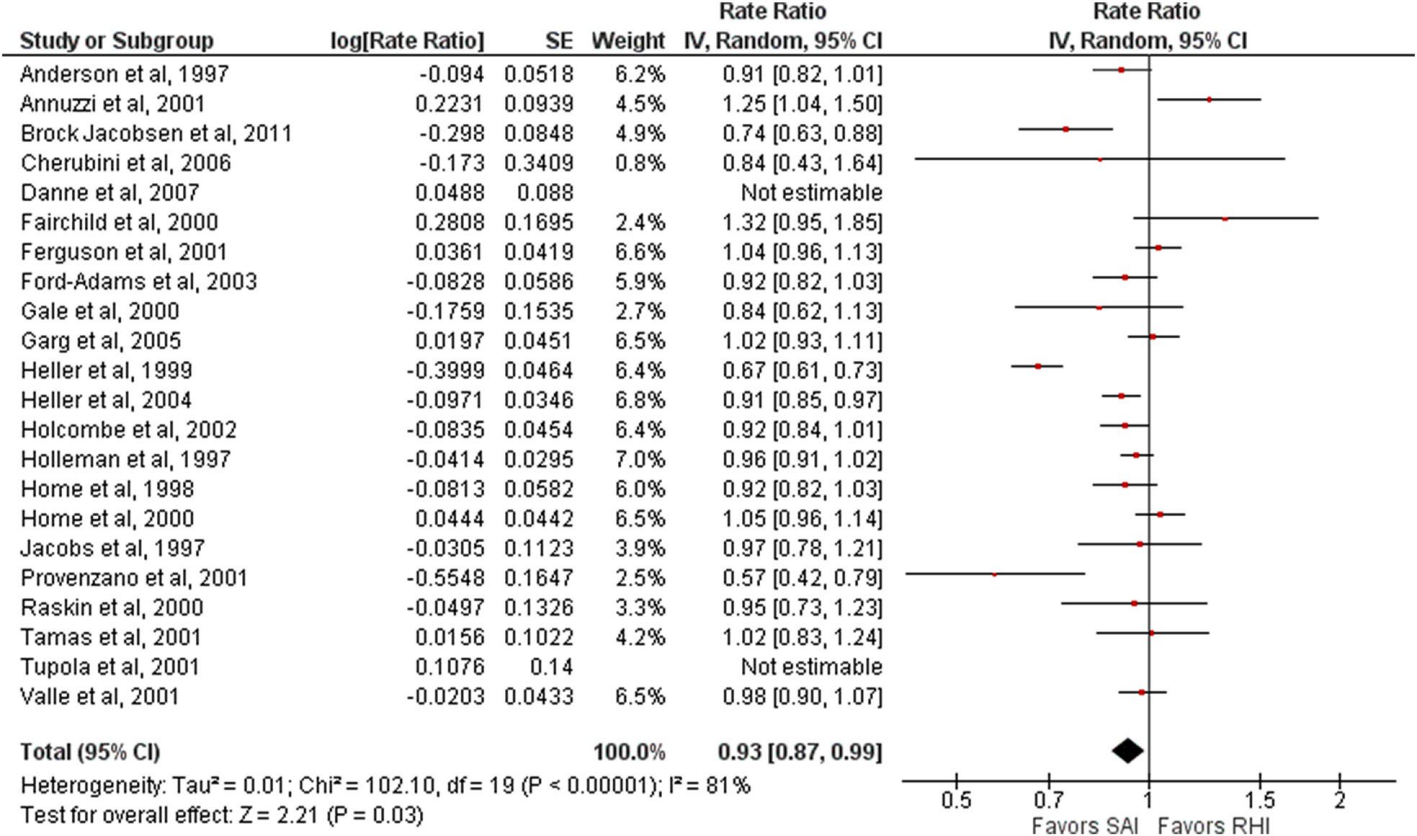
Forest plot representing all hypoglycemic episodes (for aspart, glulisine and lispro). *SAI* Short-acting insulin, *RHI* Regular human insulin

A sensitivity analysis was performed for this primary outcome (total episodes of hypoglycemia) by excluding studies with a high risk of bias [31, 41]. The results showed that short-acting insulin analogues were associated with a lower number of total hypoglycemic episodes per month when compared with regular human insulin (risk rate 0.93, 95% CI 0.87–0.99; 6180 patients, 20 studies; I^2^ = 81%) (Fig. 1). The monthly rate of total hypoglycemic episodes after the use of short-acting insulin analogues was 7% lower than in the group who used regular human insulin.

A subgroup analysis comparing the use of NPH and long-acting insulin analogues as basal insulin did not show difference between them regarding the number of total hypoglycemic episodes per month (NPH as basal insulin: risk rate 0.94, 95% CI = 0.88–1.00, 5248 patients, 18 studies; I^2^ = 80%; long-acting insulin analogues as basal insulin: risk rate 0.80, 95% CI 0.52–1.23, 902 patients, 3 studies, I^2^ = 83%).

#### Nocturnal hypoglycemia

**Fig. 2:**
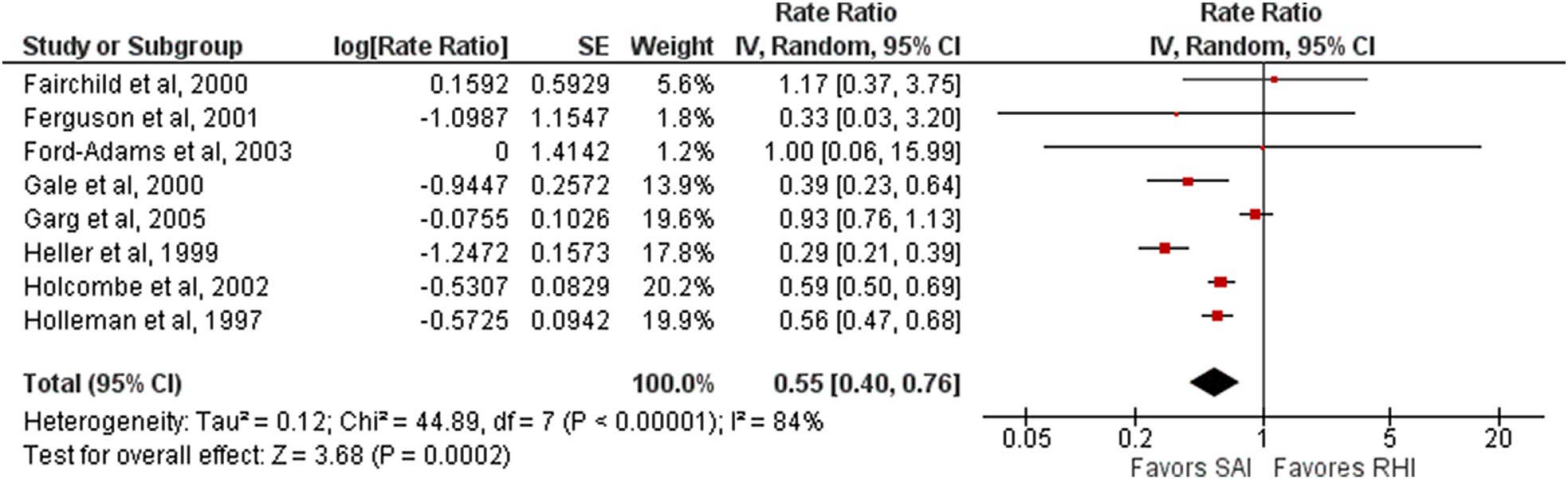
Forest plot representing nocturnal hypoglycemia (for aspart, glulisine and lispro). *SAI* Short-acting insulin, *RHI* Regular human insulin

Of the 22 studies, eight assessed episodes of nocturnal hypoglycemia, one with glulisine and seven with lispro. Their results were combined for this meta-analysis [24–28, 33, 34, 43]. In the only RCT that compared glulisine with regular human insulin, no difference was found between them (risk rate 0.93, 95% CI 0.76–1.13; 564 patients). The short-acting insulin analogues (lispro and glulisine) were associated with a 45% lower risk rate of nocturnal hypoglycemia when compared with regular human insulin (risk rate 0.55, 95% CI 0.40–0.76, 1995 patients, I^2^ = 84%) (Fig. 2).

A subgroup analysis comparing the use of NPH and long-acting insulin analogues as basal insulin showed that NPH insulin was associated with a lower number of nocturnal hypoglycemic episodes per month when compared with long-acting insulin analogues (NPH as basal insulin: risk rate 0.48, 95% CI 0.36–0.64, 1011 patients, 7 studies, I^2^ = 71%; glargine as basal insulin: risk rate 0.93, 95% CI 0.76–1.13, 860 patients, one study).

#### Severe hypoglycemia

**Fig. 3:**
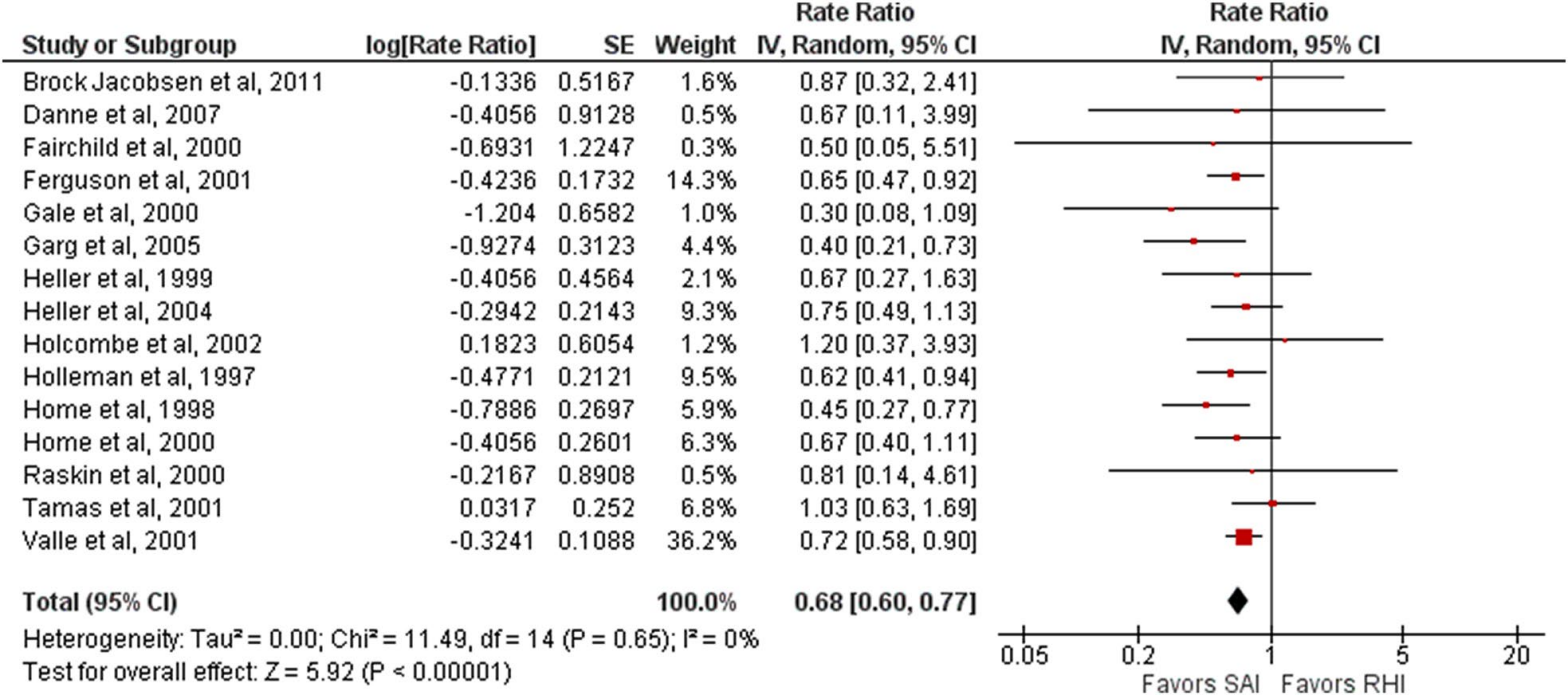
Forest plot representing severe hypoglycemia (for aspart, glulisine and lispro). *SAI* Short-acting insulin, *RHI* Regular human insulin

Of the 22 studies, 15 analyzed the episodes of severe hypoglycemia. Their results were combined for this meta-analysis [24–28, 32, 33, 35–38, 40–43]. Short-acting insulin analogues (aspart, glulisine, and lispro) were associated with a 32% lower risk rate of severe hypoglycemia when compared with regular human insulin (risk rate 0.68, 95% CI 0.60–0.77; 5945 patients, 15 studies; I^2^ = 0%) (Fig. 3).

A subgroup analysis comparing the use of NPH or long-acting insulin analogues as basal insulin showed that NPH insulin was not associated with a lower number of total hypoglycemic episodes per month when compared with long-acting insulin analogues (NPH as basal insulin: risk rate 0.70, 95% CI = 0.61–0.79, 4848 patients, 14 studies, I^2^ = 0%; glargine as basal insulin: risk rate 0.40, 95% CI 0.21–0.73, 860 patients, one study).

#### Postprandial glucose

**Fig. 4:**
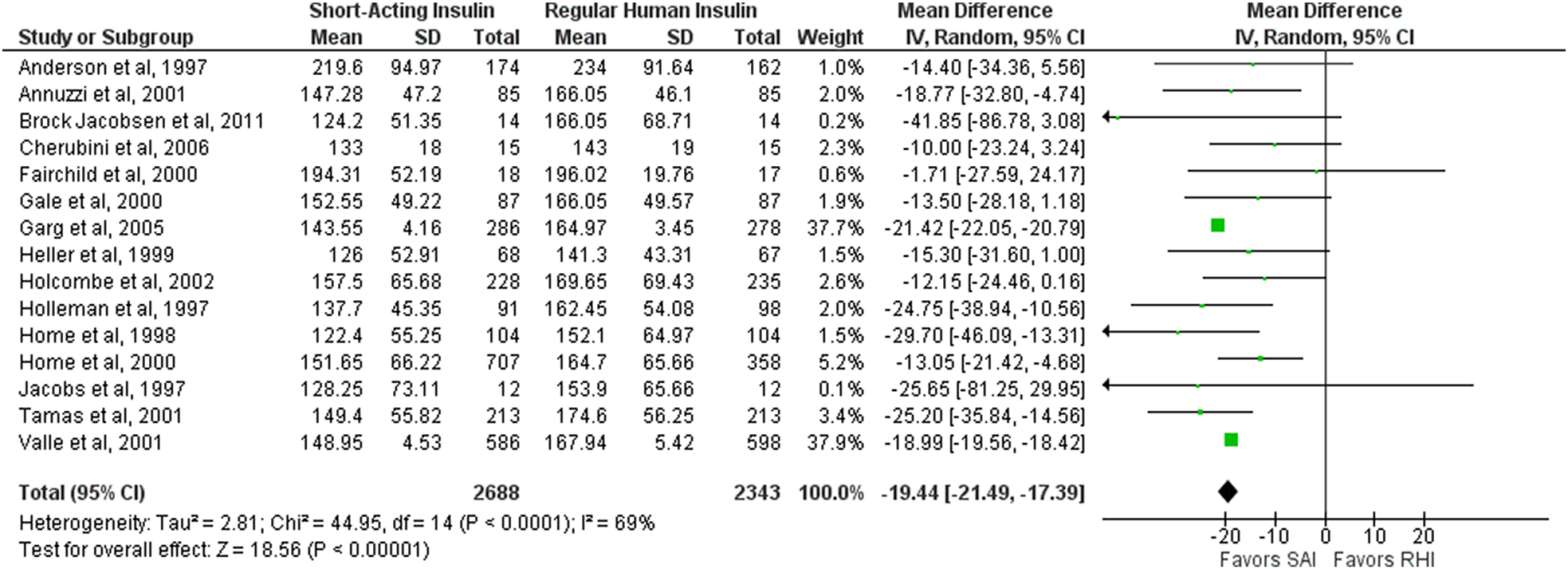
Forest plot representing postprandial glucose (for aspart, glulisine and lispro). *SAI* Short-acting insulin, *RHI* Regular human insulin

Of the 22 studies, 15 analyzed postprandial glucose (any meal). Their results were combined for this meta-analysis [22–26, 28, 29, 32, 33, 35, 36, 39, 40, 42, 43]. Short-acting insulin analogues (aspart, glulisine, and lispro) were associated with lower postprandial glucose levels when compared with regular human insulin (mean difference/MD − 19.44 mg/dL; 95% CI − 21.49 to − 17.39; 5031 patients, I^2^ = 69%) (Fig. 4).

Thirteen studies assessed postprandial glucose levels 2 h after breakfast [22–26, 28, 29, 32, 33, 36, 40, 42, 43]. We were able to pool the data from 12 studies in this meta-analysis, and the results showed that short-acting insulin analogues (aspart, glulisine, and lispro) were associated with lower postprandial glucose levels after breakfast when compared with regular human insulin (MD − 22.35 mg/dL; 95% CI − 23.52 to − 21.17; 4623 patients; I^2^ = 50%) (Additional file 5: Figure S3A).

Fourteen studies assessed postprandial glucose levels two hours after lunch. We were able to pool the data from 11 studies in this meta-analysis [23–26, 28, 29, 32, 33, 35, 36, 42], and the results showed that short-acting insulin analogues (aspart and lispro) were associated with lower postprandial glucose levels after lunch when compared with regular human insulin (MD − 10.86 mg/dL, 95% CI − 13.41 to − 8.31; 3675 patients; I^2^ = 54%). The three remaining studies [27, 30, 43] did not provide sufficient data to be pooled in this meta-analysis. Individually, none of the articles showed any difference when comparing insulin lispro with regular human insulin for postprandial glucose after lunch (Additional file 5: Figure S3B).

Fourteen studies assessed postprandial glucose levels 2 h after dinner [23, 24, 26–29, 31–33, 35, 36, 40, 42, 43]. We were able to pool the data from 12 studies in the meta-analysis, and the results showed that short-acting insulin analogues (aspart, glulisine, and lispro) were associated with lower postprandial glucose levels after dinner when compared with regular human insulin (MD − 19.52 mg/dL, 95% CI − 21.73 to − 17.31; 4530 patients; I^2^ = 90%). In the two studies not included in this meta-analysis, no difference was found between lispro and regular human insulin [27, 31]. (Additional file 5: Figure S3C).

#### Glycated hemoglobin

Nineteen studies evaluated HbA1c at the end of treatment [22–28, 30, 32–40, 42, 43]. We were able to pool the data from 15 studies in this meta-analysis, and the results showed that short-acting insulin analogues (aspart or lispro) were associated with lower HbA1c when compared with regular human insulin (MD − 0.13, 95% CI − 0.16 to − 0.10; 5204 patients; I^2^ = 73%).

When short-acting insulin analogues were assessed separately, only aspart was associated with lower HbA1c when compared with regular human insulin (MD − 0.14, 95% CI − 0.20 to − 0.02; 2822 patients, I^2^ = 40%). Lispro was not associated with lower HbA1c levels when compared with regular human insulin (MD − 0.09, 95% CI − 0.17 to 0.02; 2552 patients; I^2^ = 40%).

Four studies were not included in the meta-analysis because they did not have available data for input [23, 31, 42, 43]. The first three showed no statistically significant difference between the groups, and Jacobs et al. [23] showed a difference in favor of regular human insulin.

#### Quality of life and patient satisfaction

Five studies assessed patients’ quality of life [24, 27, 28, 36, 40]. Fergunson et al. and Gale et al. [27, 28] did not present the results, but reported no difference between the short-acting insulin analogue and regular human insulin groups. Holleman et al. [24] reported a greater flexibility in the short-acting insulin analogue group (p < 0.0001) and an even better adaptation of mealtimes (p < 0.0001), physical activity planning (p < 0.0001), and activities (p < 0.0001). Home et al. [36] used the Diabetes Treatment Satisfaction Questionnaire (DTSQ) and found a significant difference in favor of the short-acting insulin analogue group, with a score 2.3 points higher than that of the regular insulin group (95% CI 1.32 points to 3.28 points). Tamás et al. [40] also reported no difference in the overall DTSQ score, but the group that received short-acting insulin analogues reported greater flexibility of use (p = 0.022).

## Discussion

In this systematic review and meta-analysis we originally report clinical evidence on therapeutical use of short-acting insulin analogues compared with regular insulin while focusing on the main benefits of these analogues, namely the reduction of hypoglycemia and postprandial glucose levels. The combined data of 22 RCTs showed that short-acting insulin analogues are associated with a decrease in total hypoglycemic episodes, nocturnal and severe hypoglycemia, and post-breakfast, post-lunch and post-dinner glucose levels.

Fullerton et al., in a systematic review that aimed to evaluate long-term safety of short-acting insulin analogues, also assessed the outcomes described here; however, since their research was focused on long-term studies, fewer RCTs were retrieved when compared to the present study [17]. The results of these reviews cannot be compared due to the high probability of inconsistencies. Another recently-published review analyzed only trials comparing aspart with regular human insulin, but also gathered data from a smaller set of studies [44]. Since the three short-acting insulin analogues are very pharmacologically similar regarding time of onset, peak activity, and duration of action [16], analyzing data from trials conducted with only one short-acting insulin analogue yields a lower number of studies, resulting in less statistical power. We saw no clear advantage in using this approach for the current study. A third systematic review and meta-analysis described only the results of hypoglycemia, and again included a smaller number of studies. In addition, no data on postprandial glucose were reported [45].

The association between short-acting insulin analogues and a reduction of 7% in total hypoglycemic episodes, 32% in severe hypoglycemia, and 45% in nocturnal hypoglycemia levels is an important finding, as these episodes are particularly associated with lower quality of life and treatment nonadherence [46]. The pursuit of lower HbA1c levels is associated with a higher rate of hypoglycemia episodes [47], which is a well-known barrier to strict glucose control. This may hinder the maintenance of euglycemia over a lifetime, which prevents patients from fully benefitting from glucose control [48]. The benefits mentioned above are most likely determined by the specific pharmacokinetic properties of these analogues; having a very short-acting activity limits the risk of late falls in glucose levels [16]. The lower frequency of nocturnal hypoglycemic episodes observed with short-acting insulin analogues may contribute to the lower frequency of severe hypoglycemia. It is already known that sleep per se is a risk factor for severe hypoglycemia, as symptoms of hypoglycemia might be blunted or absent during sleep [8].

Short-acting insulin analogues are expected to provide more adequate insulin levels in response to increases in postprandial blood glucose, which would reflect in a better postprandial glucose control. This was observed in our meta-analysis for all postprandial glucose levels, as well as after each individual meal (breakfast, lunch, and dinner). Interestingly, even though short-acting insulin users had lower postprandial glucose levels, they also had lower frequency of hypoglycemic episodes, a double benefit brought on by the pharmacokinetics of these drugs (faster onset of action and shorter duration of action) [16].

Postprandial glucose fluctuations contribute to approximately 50% of the total hyperglycemia episodes in patients on multiple doses of insulin [49]. Therefore, short-acting insulin analogues were expected to be associated with lower HbA1c levels, which would be consistent with observed decreases in postprandial blood glucose levels. However, the decreases were clinically irrelevant, even though the short-acting insulin analogues were indeed associated with lower HbA1c levels. This could be explained by the multiple insulin regimens employed in the analyzed studies (since short-duration studies were also included), as well as by the reduction in hypoglycemic episodes. In some studies, a single dose of NPH was used as basal insulin, which is an unacceptable regimen considering the current practice aimed at strict glucose control for T1DM. It is well-known that a better metabolic control with short-acting insulin analogues can be obtained with the optimization of basal insulin [2, 14].

Limited evidence analyzed in this systematic review suggests that, for patients with T1DM, the treatment with short-acting insulin analogues is more convenient than with regular human insulin. The higher satisfaction levels and greater flexibility attributed to short-acting insulin analogues could be explained by the fact that they can be administered immediately before meals, as opposed to the anticipated 30 to 45 min when administering regular human insulin. In a study involving 1184 patients with T1DM, adherence to the correct timing of regular human insulin was 7% for patients who took it more than 30 min before meals, 60% for those who took it 15-30 min before meals, and 33% for those who took it 15 min before meals. Regarding the administration of insulin lispro, 98% of the patients followed the orientation (0 to 15 min before meals) [32]. The possibility of administration of short-acting insulin analogues immediately after meals is another important benefit, as it may not always be possible to predict how much food (carbohydrates) the patient will have eaten at the end of the meal.

Due to the scarcity of studies assessing the impact of short-acting insulin analogues on the quality of life of patients with T1DM and the methodologies used, previously-published systematic reviews either did not analyze this outcome or did not reach a conclusion [50]. According to Fullerton et al. [17], with a more adequate methodology (the DTSQ) [51], three studies reported no improvement in treatment satisfaction, while four studies indicated an improvement in this outcome with short-acting insulin analogues when compared to regular human insulin.

As opposed to other meta-analyses [17], this review provided information regarding the use of insulin analogues in children. However, no association was found between the use of short-acting insulin analogues or regular human insulin and the number of hypoglycemic episodes, postprandial glucose reductions, and HbA1c, probably because of the low number of studies included.

The main methodological strengths of this review are as follows: the most adequate outcomes considering the pharmacokinetics of short-acting insulin analogues; the most comprehensive and systematic literature search among systematic reviews on this subject, with no language restriction; and the specific and reproducible eligibility criteria, study selection, and data extraction.

However, some limitations should be pointed out. The first is that most studies included in our systematic review may not represent current T1DM treatment practice. Most trials excluded patients with hypoglycemia unawareness or with a high risk of hypoglycemia, which in fact makes up the largest population group that could benefit from insulin analogues in the current clinical practice. Additionally, the low quality of most studies identified in this systematic review may limit the interpretation of the presented data. The differences in the definition of total and nocturnal hypoglycemic episodes, as well as the methods for recording hypoglycemic episodes based on the presence of symptoms or on the obligatory verification of blood glucose independently of symptoms, are real limitations frequently observed in clinical trials. Another limitation is the absence of masking, which could also result in a high risk of bias. However, it is unlikely that future studies will adequately mask the participants, as this would require a significant increase in the number of insulin applications. The analyses with NPH as basal insulin included 7, 18 and 14 studies (nocturnal hypoglycemia, total hypoglycemia and severe hypoglycemia, respectively), and those with long-acting insulin analogues included 1, 3 and 1 studies (nocturnal hypoglycemia, total hypoglycemia and severe hypoglycemia, respectively), and these analyses presented high heterogeneity, precluding their consideration as a definitive evidence of the possible superiority of NPH as compared to long-acting insulin analogues. This information should, thus, be interpreted with caution. A direct comparison between NPH insulin and long-acting analogues is beyond the scope of this review. Another important point is that, over the years, there has been a significant evolution in insulin therapy, which can be observed in the clinical heterogeneity between studies in the past 20 years.

In summary, short-acting insulin analogues were associated with fewer nocturnal and severe hypoglycemic events and better glucose control (slightly lower HbA1c and lower postprandial blood glucose levels) when compared with regular human insulin in subjects with type 1 diabetes.

## Additional files

**Additional file 1: Table S1.** Search strategy applied to all databases.

**Additional file 2: Figure S1.** Flow diagram: identification and selection of articles included in the meta-analysis.

**Additional file 3: Figure S2.** Percentage distribution of risk of bias by domain.

**Additional file 4: Table S2.** Characteristics and Quality Of Studies.

**Additional file 5: Figure S3.** Forest plot representing postprandial glucose for breakfast (A), lunch (B), and dinner (C) (for aspart, glulisine and lispro). *SAI: Short*-*Acting Insulin; RHI: Regular Human Insulin*.

**Additional file 6: Table S3.** Analysis of risk of bias of the selected studies.
