# Supplementary figures and images for: Short-acting insulin analogues versus regular human insulin on postprandial glucose and hypoglycemia in type 1 diabetes mellitus: a systematic review and meta-analysis
Source: Diabetol Metab Syndr. 2019 Jan 3;11:2. doi: 10.1186/s13098-018-0397-3 (PMC6317184; doi:10.1186/s13098-018-0397-3)

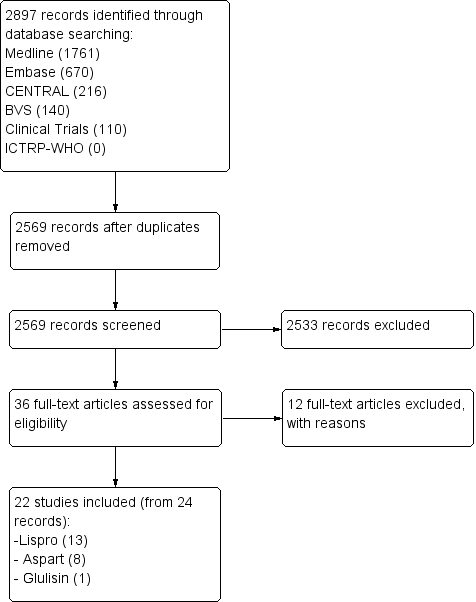

Supplement: Supplementary file 2 — Additional file 2: Figure S1. Flow diagram: identification and selection of articles included in the meta-analysis. [file 13098_2018_397_MOESM2_ESM.png]

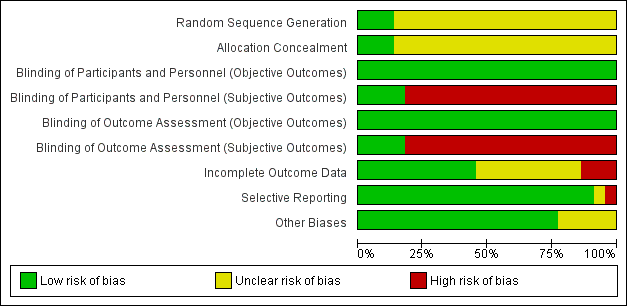

Supplement: Supplementary file 3 — Additional file 3: Figure S2. Percentage distribution of risk of bias by domain. [file 13098_2018_397_MOESM3_ESM.png]

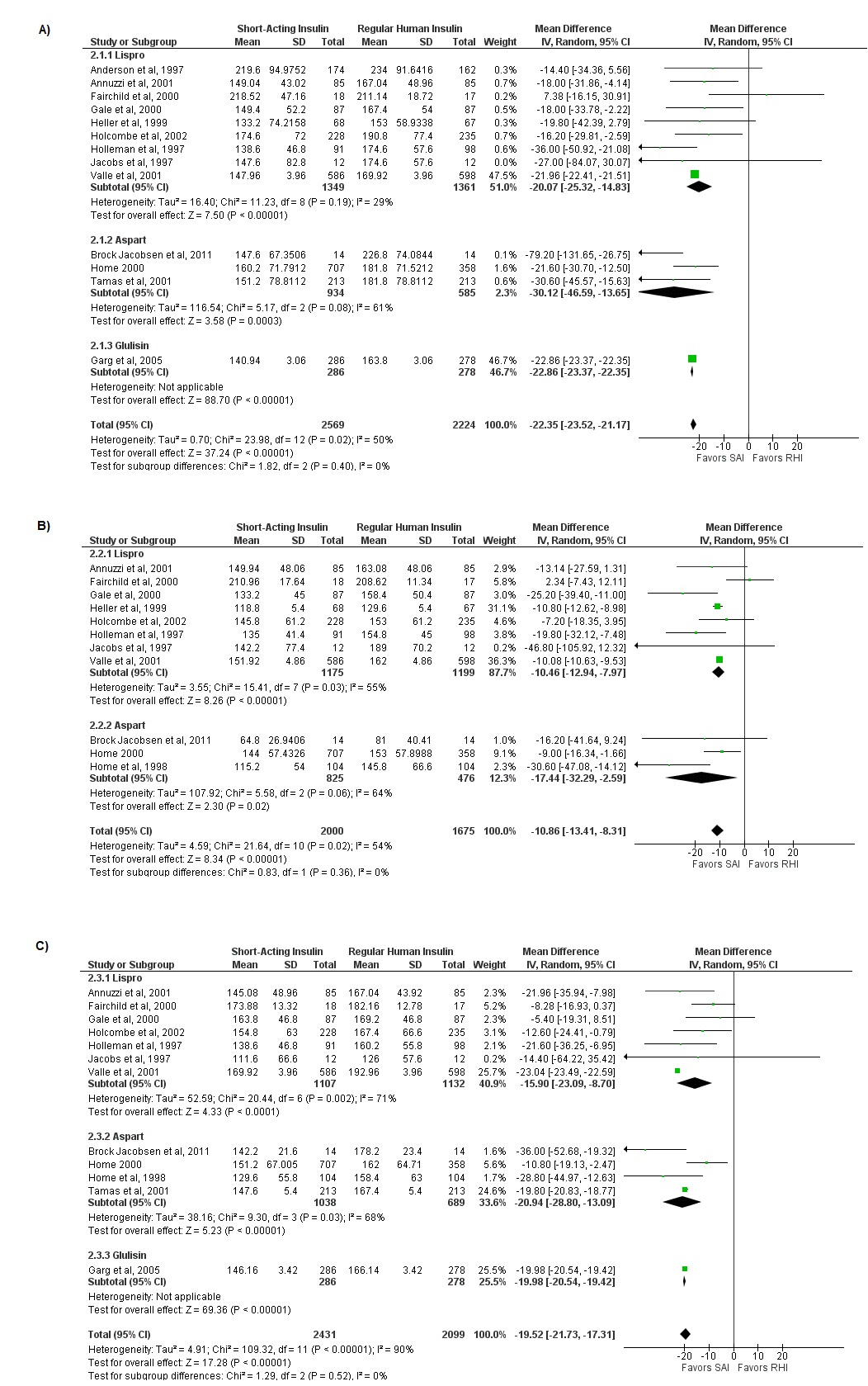

Supplement: Supplementary file 5 — Additional file 5: Figure S3. Forest plot representing postprandial glucose for breakfast (A), lunch (B), and dinner (C) (for aspart, glulisine and lispro). SAI: Short-Acting Insulin; RHI: Regular Human Insulin. [file 13098_2018_397_MOESM5_ESM.png]
